# From Structure to Functional Implications: Investigation of the Melon-Like Framework of Graphitic Carbon Nitride for Li–S Batteries

**DOI:** 10.1021/acsomega.5c06720

**Published:** 2025-09-25

**Authors:** Jyoti Pandey, Aliakbar Yazdani, Mukesh Jakhar, Valeri Petkov, Veronica Barone, Chi-Hao Chang, Gabriel Caruntu, Yi Ding, Bradley D. Fahlman

**Affiliations:** † Department of Physics, 5649 Central Michigan University, Mt. Pleasant, Michigan 48859, United States; ‡ Department of Chemistry and Biochemistry, Central Michigan University, Mt. Pleasant, Michigan 48859, United States; § Inter-University Accelerator Center, Aruna Asaf Ali Marg, New Delhi 110067, India; ∥ The Dow Chemical Company, Dow Performance Silicones, 5300 11 Mile Road, Auburn, Michigan 48611, United States; ⊥ U.S. Army Combat Capabilities Development Command (DEVCOM)—Ground Vehicle Systems Center (GVSC), Warren, Michigan 48397, United States

## Abstract

Graphitic carbon nitride (gCN) has emerged as a promising material for sustainable energy storage applications, photocatalysis, and sensors. Despite extensive research, its precise crystallographic structure remains controversial, particularly regarding the distinction between heptazine-based and melon-like frameworks. In this study, we investigate the structural characteristics of gCN synthesized using different precursors, urea and melamine. Comprehensive characterization, including X-ray diffraction (XRD), confirms that all samples exhibit a melon-like framework with *P*2_1_2_1_2 symmetry. This contradicts the widely cited but experimentally unsupported heptazine-based model. We further explore how precursor selection influences crystallinity, elemental composition, and electrochemical behavior. Additionally, density functional theory (DFT) studies are also incorporated to support the experimental findings. Notably, our study is among the first to report the electrochemical performance of gCN with a confirmed melon-like structure, highlighting the strong correlation between structural attributes and functional properties. These findings provide valuable insights into the structure–property relationships in gCN and open new avenues for its rational design and applications in energy storage and conversion technologies for metal–sulfur batteries.

## Graphical abstract

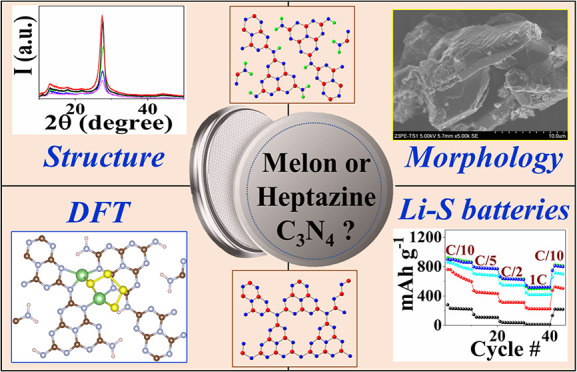

## Introduction

The pursuit of efficient and sustainable energy storage materials has led to significant interest in exploring graphitic carbon nitride (gCN).^1,2^ Due to its unique layered structure, high nitrogen content, and semiconducting properties, gCN offers potential applications in energy storage, photocatalysis, and sensors.^3–8^ Graphitic carbon nitride has also been a hot topic among researchers due to its tunable electronic structure and chemical stability.^9^

The graphitic phase is regarded as the most stable phase under ambient conditions out of the numerous allotropes of carbon nitrides.^10–12^ The first report for synthesizing a polymeric carbon nitride dates back to 1834 by Berzelius and Liebig.^13^ It adopts a polyconjugated network comprised of carbon and nitrogen atoms and is characterized by a layered graphitic-like structure.^14^ The fully polymerized form of gCN should have a C/N ratio of 0.75, which is almost impossible to obtain practically. This results in the presence of 1 to 2% hydrogen, depending on the synthetic procedure.^15^

There exists a number of graphitic carbon nitride (g-C_*x*_N_*y*_) materials that are typically reported to be wide-gap semiconductors (band gap ∼2.4–2.7 eV).^14,16^ The investigation of the crystal structure of these materials dates back to the early 20th century.^17–19^ Since then, two two-dimensional (2D) structures have been proposed—the fully condensed graphitic carbon nitride, tri-s-triazine (heptazine, C_6_N_7_), and s-triazine (C_3_N_3_), with the polymerized heptazine layered structures predicted to be the most thermodynamically stable forms.^11,20–28^ In 2009,^29,30^ the 2D structure of gCN (synthesized by thermal condensation) was confirmed as a tri-s-triazine-based polymeric structure with the help of studies such as electron diffraction,^29,30^ and solid-state nuclear magnetic resonance.^29^ However, since the material had a highly disordered behavior, a detailed explanation of the three-dimensional (3D) structure had only been suggested^31^ rather than experimentally obtained. Bojdys et al.^32^ proposed a salt-melt synthesis (SMS) of gCN as an alternative to the conventional thermal condensation. A uniform crystalline sample was obtained; however, the crystal model proposed by Bojdys et al. did not match the diffraction patterns, possibly because it was fitted under the assumption that the sample was free from any potential impurities such as hydrogen, chlorine, or lithium, which are the constituent elements of the precursors for the SMS synthesis of gCN. Schnick and co-workers^33^ proposed a lamellar structure with the intercalation of LiCl, although their results did not reproduce the X-ray diffraction (XRD) results. Bojdys and co-workers^34^ mentioned that triazine-based gCN could be detected at the gas–liquid and solid–liquid interfaces in the reactor of SMS, which suggests that SMS syntheses can promote the production of gCN with triazine units. However, the crystal structure of the detected gCN was not determined because of a lack of observable XRD peaks for bulk gCN. Due to these challenges, the structural nature of gCN remains a topic of debate.

Fina et al. explored the structure explicitly using XRD and neutron diffraction techniques.^35^ Their report supports a melon-like polymeric structure for gCN over a heptazine-based framework with the crystallographic symmetry *P*2_1_2_1_2.^35^ To the best of our knowledge, the XRD patterns of all the experimentally synthesized graphitic carbon nitride that are available so far in the literature match with the melon-like framework with *P*2_1_2_1_2 symmetry. Despite this agreement, it is widely claimed that the structure corresponds to a heptazine-based framework. This structural ambiguity has significant implications on the electronic properties, defect chemistry, and catalytic activity of gCN, thus necessitating further systematic studies. To address these issues, we selected one of many approaches, namely, choice of precursors, such as urea and melamine, which influence structural parameters such as crystallinity, defect formation, and elemental composition. Interestingly, all the synthesized samples, along with a commercial sample, featured a melon-like framework. This study also highlights the impact of precursor-dependent synthesis and post-treatment on the physicochemical and electrochemical properties of gCN, offering a pathway for optimizing its functionality in energy storage and conversion applications. To the best of our knowledge, we are the first to report the electrochemical properties of graphitic carbon nitride for metal–sulfur batteries, considering its structure as a melon-like framework. The combination of experimental and computational studies provides a deeper understanding of structure–property relationships in gCN-based materials, contributing to the ongoing efforts to develop advanced carbon nitride-based materials for sustainable energy technologies.^7,36^

## Experimental Section

### Material Synthesis and Characterization

#### Synthesis of Pristine Graphitic Carbon Nitride (_p_CN) Samples

_p_CN samples were synthesized by using urea (99%, Fisher Scientific) and melamine (99%, Sigma-Aldrich) as precursors. In a typical synthesis, urea and melamine were separately placed in covered alumina crucibles and heated in a muffle furnace at 550 °C for 4 h under an ambient atmosphere. The heating rate was maintained at 2.5 °C min^–1^. The samples were naturally cooled to room temperature. The obtained yellowish powders were designated as u_p_CN (synthesized from urea) and m_p_CN (synthesized from melamine), respectively.

#### Thermal Reduction of _p_CN Samples

The pristine CN (_p_CN) samples were further thermally reduced under vacuum conditions. The reduction was carried out in a tube furnace at 590 °C for 4 h at a heating rate of 3 °C min^–1^. The process was conducted under a continuous vacuum to enhance the removal of residual species and further modify the structural properties. The thermally reduced samples were designated as u_r_CN (synthesized from urea) and m_r_CN (synthesized from melamine), respectively.

In addition, gCN was also purchased from ACS Materials (99%) to compare the structural and electrochemical properties.

#### Characterization Techniques

Powder X-ray diffraction (PXRD) patterns of the samples were recorded by using a Rigaku diffractometer operated at 45 kV and 250 mA over the 2θ range of 10–50°. The XRD measurements for the semi-in situ electrochemical studies were performed using a Panalytical diffractometer using Mo Kα radiation and a secondary monochromator to record high-resolution Bragg peaks. Profile matching of the PXRD patterns was performed by using the Crystal Diffract software. Morphological analyses of the samples were performed using a Hitachi 3400N–II scanning electron microscope equipped with a tungsten filament electron gun and a large, motorized specimen chamber. Raman spectra were collected with a Horiba Jobin Yvon Xplora Raman Spectrometer using a 100× microscope objective and a 532 nm excitation of an Nd/YAG laser with a maximum power of 15 mW. The measurements were performed with a 10 mm pinhole and a spectral resolution of 1 cm^–1^. The laser spot size was 2 mm, and the collected data were analyzed with Labview 6 software provided by Horiba to precisely locate the Raman bands. Brunauer–Emmett–Teller (BET) surface area measurements were performed using the adsorption–desorption method with an automated surface area and pore size analyzer [Autosorb IQ Asiqwin Version 5.2x Model 7 (Anton Paar, USA)]. Elemental analyses of the samples were performed using the Alfa Chemistry testing lab (Agilent ICP-OES 730 and Thermo Flash 2000). X-ray photoelectron spectroscopy (XPS) measurements of the samples were recorded on a Kratos Axis Supra+ spectrometer equipped with monochromatic Al Kα radiation. The core-level spectra were calibrated by considering the C 1s core level as the standard. The obtained raw spectra were fitted by using XPSPEAK 4.1 program.

#### Computational Studies

Spin-polarized DFT calculations were carried out using the Vienna Ab initio Simulation Package.^37,38^ The exchange-correlation interaction was described by the Perdew–Burke–Ernzerhof (PBE) functional^39^ within the generalized gradient approximation, supplemented by the DFT-D3 semiempirical correction to account for long-range van der Waal (vdW) interactions.^40^ A plane-wave cutoff energy of 520 eV was applied, and the projector augmented-wave (PAW) method was utilized for electron–ion interactions.^41^ Periodic boundary conditions were considered with a vacuum of 20 Å to minimize the interactions between neighboring cells along the *c*-direction. The Brillouin zones were sampled using 3 × 3 × 1 γ-centered Monkhorst–Pack *k*-point meshes for the geometry optimization of a 2 × 2 supercell of g-C_3_N_4_. During the geometric optimization of lithium polysulfides (LiPSs) adsorbed on the substrates, the atomic positions were fully relaxed while the cell size was kept fixed until the residual force fell below 0.01 eV/Å. The climbing image nudged elastic band (CI-NEB) method was employed to calculate the energy barriers of Li_2_S decomposition.^42,43^

The adsorption energies (*E*_ads_) of LiPSs on the gCN substrate were evaluated using the following equation^44,45^

1 $$E_{\mathrm{ads}}=E_{\mathrm{LiPSs}}+E_{s\mathrm{ubstrate}}-E_{\mathrm{total}}$$

Where *E*_total_, *E*_substrate_, and *E*_LiPSs_ represent the energies of the LiPS molecule adsorbed on the gCN substrate, the isolated gCN substrate, and the isolated LiPS molecule, respectively. Based on this definition, a positive *E*_ads_ indicates stable binding between the molecule and the substrate.

We examined the energetic stability of the structures by calculating the formation energy (*E*_form_) of the considered melon and crystalline gCN monolayers:

2 $$E_{\mathrm{form}}=\frac{\left(E_{\mathrm{substrate}-n_{c}E_{c}}-n_{N}E_{N}-n_{H}E_{H}\right)}{n_{\mathrm{tota}l}}$$

where *E*_form_ represents the formation energy of gCN, *E*_substrate_ denotes the total energy of crystalline or melon gCN unit cell, *E*_C_ and *E*_N_, and *E*_H_ correspond to the total energies per atom of carbon, nitrogen, and hydrogen, extracted from the graphene monolayer, N_2_, and H_2_ molecules, respectively. The calculated formation energies of 2D crystalline and melon gCN are 0.26 and −0.039 eV/atom, respectively. These values indicate that the melon structure is more stable than its already stable constituents, while the heptazine gCN is metastable.

### Electrochemical Measurements

#### Synthesis of Carbon–Sulfur Composites

The synthesized pristine and reduced carbon hosts (_p_CN and _r_CN, respectively) were homogeneously mixed by using an agate mortar and pestle. Analytical-grade precipitated sulfur (Putratonic, ≥99.99%, Alfa Aesar) was added at a weight ratio of 75% sulfur and 25% gCN in the final composite. The mixture was transferred into a Teflon-lined hydrothermal reactor and heated at 155 °C for 12 h to facilitate sulfur infiltration into the mesopores of the carbon hosts via melt-diffusion. The resulting carbon–sulfur (C–S) composite was stored under an inert atmosphere until further characterization.

#### Coin-Cell Assembly

All electrolyte preparations and coin-cell assembly were conducted in an argon-filled glovebox with the concentrations of the O_2_ and H_2_O maintained below 1 ppm.

For the electrolyte, 2.87 g of LiTFSI (anhydrous purity ≥ 99%, SynQuest Laboratories) was dissolved in 10 mL of an ether-based solvent (1:1 mixture of DME (anhydrous purity ≥ 99%, Sigma-Aldrich) and DOL (anhydrous purity ≥ 99%, Sigma-Aldrich)). Two wt % of LiNO_3_ (anhydrous, ≥99%, Sigma-Aldrich) was added as an electrolyte additive to enhance the stability of the lithium metal anode. The solution was stirred overnight to ensure the complete dissolution of all components.

Cathodes were prepared by using a slurry-casting method. The cathode slurry consisted of the active material (C–S composite), carbon black (Timcal Super C65, MTI Corp), and PVDF binder (grade HSV900, Kynar) in a weight ratio of 7:2:1, dispersed in NMP (anhydrous purity ≥ 99%, Sigma-Aldrich) solvent. The slurry was transferred into a 20 mL ZrO_2_ grinding bowl along with 8 ZrO_2_ balls and ball-milled at 900 rpm for 30 min to achieve a homogeneous mixture.

The homogeneous slurry was coated onto an aluminum current collector (thickness of 15 μm, sourced as a battery cathode substrate) using an automatic electrode casting machine, producing an initial electrode thickness of 100 μm. The coated electrodes were predried on the casting bed at 70 °C for 15 min, followed by vacuum drying overnight at 50 °C. The dried electrodes were punched into circular disks with a diameter of 16 mm using a precision punch. The mass of the active material and the areal loading (1.4–1.8 mg/cm^2^) were determined using a Mettler-Toledo microbalance.

The punched cathode disks were transferred into a glovebox for final coin-cell fabrication. CR2032-type coin cells (stainless steel, MTI Corp) were assembled using punched cathode disks, a lithium metal disc (thickness of 0.6 mm and diameter of 16 mm, MTI Corp.) as the anode, a microporous polypropylene separator (Celgard, USA), and 80 μL of the prepared electrolyte applied using an autopipet to maintain a consistent sulfur-to-electrolyte ratio.

The fabricated coin cells were galvanostatically cycled (charge–discharge) at various current densities (C-rates) within the voltage window of 1.7–2.8 V vs Li/Li^+^ using a Maccor series 4600A automatic battery tester. The specific capacities were calculated based on the mass of active sulfur in the cathode. The C-rates were defined with 1C = 1672 mAh g^–1^, corresponding to the theoretical capacity for the complete electrochemical reduction of sulfur to Li_2_S.

## Results and Discussion

The XRD patterns of as-synthesized graphitic carbon nitrides (m_p_CN, m_r_CN, u_p_CN, and u_r_CN) and commercial graphitic carbon nitride (CN) samples are displayed in Figure 1a. All the samples exhibit strong diffraction peaks at 27.4° (002) and weaker peaks at 12.9° (100), indicating layered structures.^14,25^ The strong peak located at 2θ ∼ 27.4° with a *d* spacing of around 0.32 nm is attributed to the periodic stacking of the conjugated aromatic layers.^46^ Among all samples, m_p_CN has the highest intensity peak, indicative of a finer adjustment of the aromatic planes due to more complete condensation.^14^ The crystallinity of the mCN samples appeared to be comparable to the CN samples and better than the uCN samples. There is a small reflection peak located at 2θ ∼ 13° with an in-planar repeat period of 0.68 nm, which can be ascribed to the distance between adjacent pores.^47^ This peak becomes more pronounced as we move from urea-derived (uCN) to melamine-derived (mCN) graphitic carbon nitride, likely because mCN samples have better in-plane organization and more structural motifs for gCN to be connected, resulting in a larger planar size. We observe a few more peaks at 2θ ∼ 18°, corresponding to (310) and (10) planes, caused by the stacking of melon sheets.^29^ The diffraction peaks of mCN samples are stronger and sharper than those of uCN, revealing the presence of larger crystalline domains. One of the other reasons for the low polymerization of uCN samples could be the structure of the urea [C­(O)­(NH_2_)_2_]. The strong electronegativity of the O atom, as well as the strong C–O bond, is known to lead to lower polymerization^48^ than melamine-derived (C_3_N_6_H_6_) samples, which lack oxygen atoms in the precursor structure. The weaker intensity peaks of the _r_CN samples with respect to their _p_CN samples are suggestive of a decrease in the order of filling of the in-plane structure due to more defects and decreased thickness of _r_CN caused by pressure-thermal dual driving forces during the thermal reduction process. A thorough investigation of structural arrangements was performed using various crystallographic information available in the literature. Numerous compositions and models exist in the literature that explain the different structural arrangements of graphitic carbon nitride.^11,20,49–51^ Figure S1 (See Supporting Information) shows the comparison of our XRD results with different structural models with different symmetries and compositions. As can be seen in Figure 1b, irrespective of the method and precursor used for synthesis, the resultant graphitic carbon nitride always results in a melon-like structure with *P*2_1_2_1_2 symmetry.^35^

**Figure 1:**
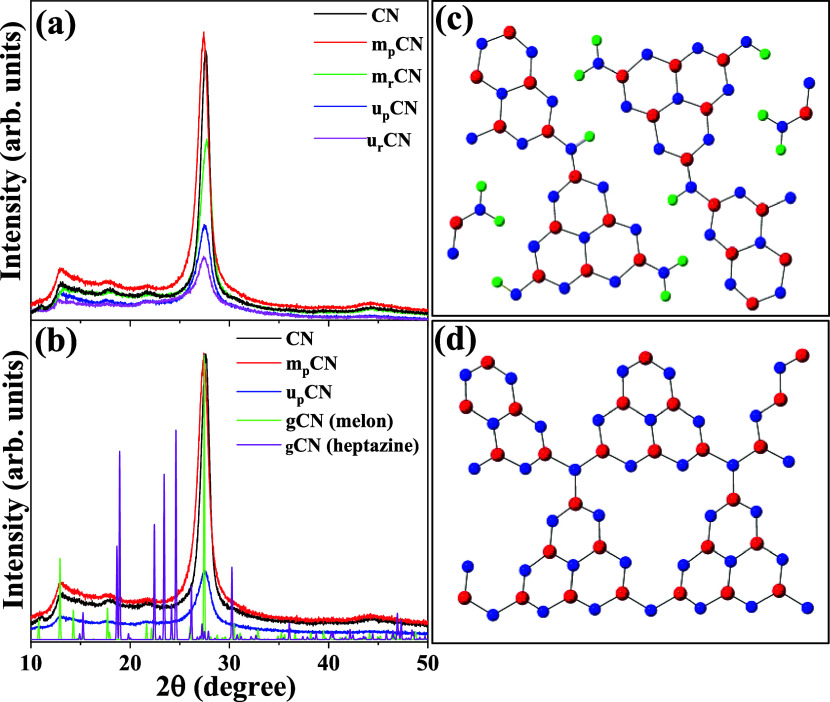
(a) XRD patterns of pristine and reduced graphitic carbon nitride synthesized using different precursors (urea and melamine) and commercial graphitic carbon nitride (CN). (b) Comparison of the XRD results for pristine graphitic carbon nitride (both melamine and urea) and commercial graphitic carbon nitride with the melon-like structure from literature^35^ and with computed data from the structure simulated by DFT considering the heptazine model. (c) and (d) Projections of the crystal structures of gCN with *P*2_1_2_1_2 symmetry^35^ and computed data corresponding to the heptazine model, respectively. C, N, and H are represented in red, blue, and green, respectively. Abbreviations- CN: commercial graphitic carbon nitride, m_p_CN: melamine-based pristine graphitic carbon nitride, m_r_CN: melamine-based reduced graphitic carbon nitride, u_p_CN: urea-based pristine graphitic carbon nitride, u_r_CN: urea-based reduced graphitic carbon nitride, gCN (melon): gCN with *P*2_1_2_1_2 symmetry, and gCN­(heptazine): heptazine model simulated by DFT.

Additionally, we also investigated the energetics of the two structural models, viz. melon-like and heptazine, using density functional theory (DFT) calculations. Based on formation energy calculations, the melon-like structure is more stable than the heptazine structure. The strong agreement between the experimental and simulated patterns for the melon-like structure with *P*2_1_2_1_2 symmetry confirms its predominance. While the heptazine-based configuration is thermodynamically feasible, it does not reproduce the experimentally observed diffraction peaks, further reinforcing the reliability of the melon-like model. Both pristine and reduced structures of heptazine and melon-like models were subjected to simulations for thoroughly understanding the sites. As shown in Figure S3 (See Supporting Information), reduced gCN structures were modeled by introducing nitrogen (N) and hydrogen (H) atom vacancies for both the heptazine and melon-like models (hg-CN and mg-CN, respectively). In the reduced configurations, the selected N and H atoms were chosen based on previous studies, which identified them as having the lowest formation energy, suggesting that these vacancies are the most likely to form.^52,53^

Raman spectroscopy is a powerful technique to explore the structural features of graphitic materials. Carbon-based graphitic materials are primarily characterized by two Raman bands related to the graphitic carbon (G band, arising from the bond-stretching motion of pairs of sp^2^ C atoms, in aromatic rings or olefinic chains) and the disordered carbon (D band, arising from the breathing modes of sp^2^ atoms in clusters of 6-fold aromatic rings),^54,55^ located at ∼1600 and 1350 cm^–1^ respectively (Figure 2a). The G and D bands correspond to the in-plane bond-stretching motion of carbon atoms in sp^2^ configuration with E_2g_ symmetry, and a breathing mode with A_1g_ symmetry, which is forbidden in pristine graphite and becomes active in disordered graphite-like structures, respectively. The Raman shift of these bands is often difficult to observe visually. To access this, the differences in the intensity of D and G bands are compared for disorder/defect evaluation. For carbonaceous materials exhibiting a highly disordered structure with the atoms that are not 100% sp^2^ bonded, the Three-Stage Model (TSM) can be used to provide an alternative, allowing correlation of values of *I*_D_/*I*_G_ to the sp^2^/sp^3^ bonding ratio.^56–58^ The intensity ratio of the two peaks (i.e., *I*_D_/*I*_G_) can be correlated to the defect density and the size of the sp^2^ C cluster in the material.^55^ Since the *I*_D_/*I*_G_ ratio is directly proportional to the defect density in the system, a higher ratio indicates the development of defect sites in the network.

**Figure 2:**
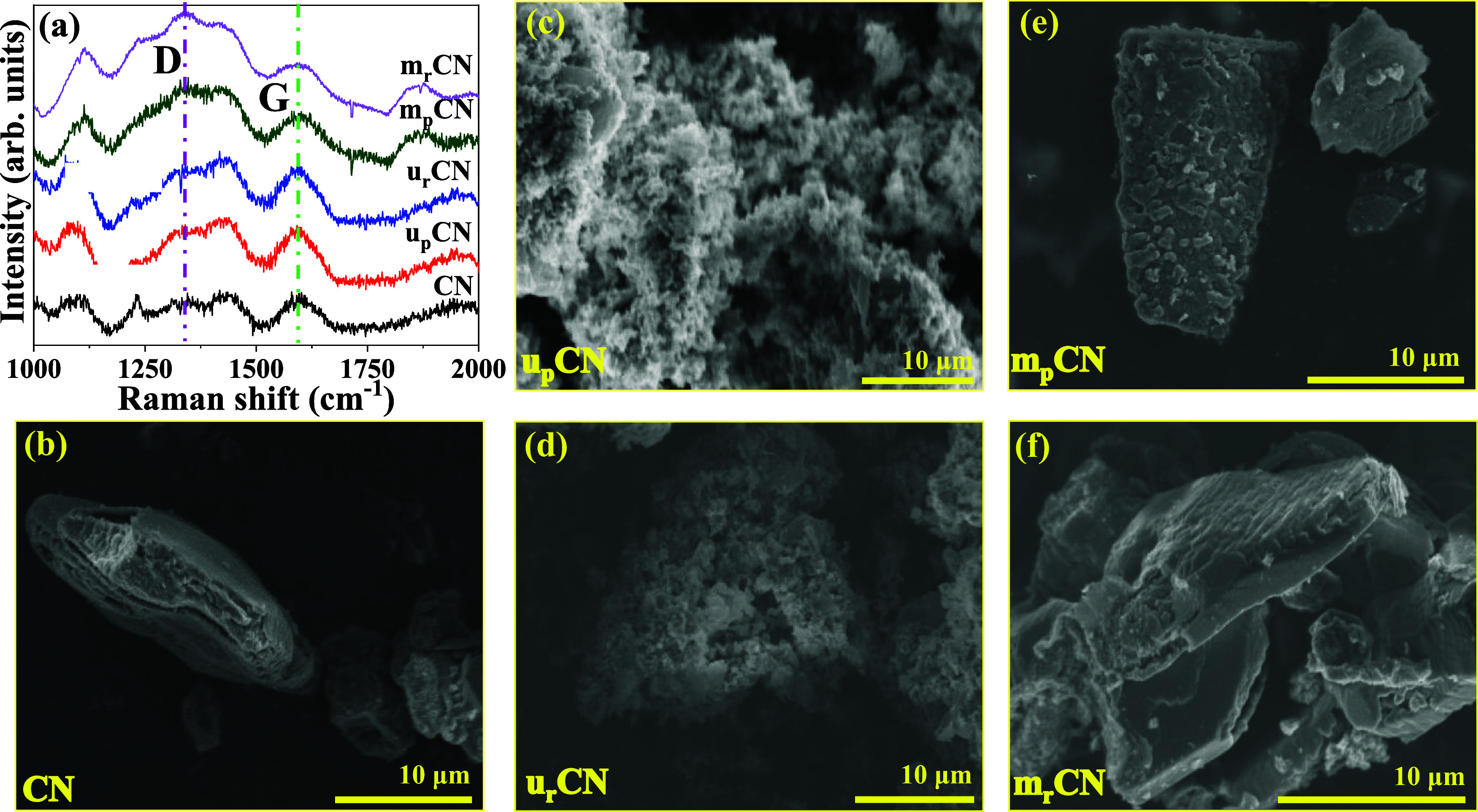
(a) Raman spectra of the gCN samples (before and after reduction) along with commercially purchased gCN. The D (defect) and G (graphitic) bands are highlighted with pink and green dotted lines, respectively. SEM images of (b) CN, (c) u_p_CN, (d) u_r_CN, (e) m_p_CN, and (f) m_r_CN. Samples synthesized from urea show an amorphous morphology, whereas the commercial and melamine-based gCN appear to be more crystalline.

As can be seen from Table 1, the *I*_D_/*I*_G_ ratio increases for the reduced samples. The highest value is observed for the reduced melamine (m_r_CN) sample, which corresponds to the development of more defect sites in the network. In addition, the removal of attached −OH and −NH groups takes place during reduction, which results in a larger size of sp^2^ C–N clusters.^55^ This is supported by the presence of a lower oxygen content in samples with a higher *I*_D_/*I*_G_ ratio. Relevant to battery applications, a lower oxygen content generally enhances conductivity, allowing for better electron transport during electrochemical reactions.

**Table 1:** BET Surface Area, Pore Diameter, Pore Volume, Elemental Composition, and Intensities of Defect (D) and Graphitic (G) Bands from Raman Spectra for the Different Graphitic Carbon Nitride Samples

sample	BET surface area (m^2^/g)	pore diameter (nm)	pore volume (cc/g)	elemental composition	*I*_D_/*I*_G_
CN	87	3.072	0.018	C_3_N_5.59_H_0.15_O_0.12_	0.83
m_p_CN	89	3.069	0.019	C_3_N_5.43_H_0.15_O_0.12_	1.05
m_r_CN	91	3.074	0.022	C_3_N_5.36_H_0.13_O_0.089_	1.08
u_p_CN	154	3.467	0.196	C_3_N_5.22_H_0.15_O_0.81_	0.94
u_r_CN	188	3.422	0.182	C_3_N_5.20_H_0.12_O_0.26_	1.01

The morphological studies show the formation of better crystallinity in the mCN samples relative to that of uCN samples. The BET N_2_ adsorption and desorption and pore size distribution curves of different gCN samples are shown in Figure S2 (See Supporting Information). All of the isotherms exhibit a type IV isotherm, and the pore size distribution indicates that the samples are mesoporous. Urea-based gCN samples are described as composed of aggregates of small crystallites, giving rise to an amorphous morphology. Due to this, uCN samples tend to have higher surface areas than mCN and CN samples (Table 1). For battery applications, proper control over the specific surface area and the corresponding pore size of materials is also crucial since high microporosity can lead to a dramatic drop in capacity at a large current density. If the material has a good intrinsic conductivity or a conductive network, it can facilitate efficient electron transport, which can compensate for the lower surface area. In materials such as gCN, stable active sites that resist degradation over repeated cycles contribute to better capacity retention.

To gain deeper insights into the bonding nature and speciation of C and N, XPS analyses were conducted to investigate the synthesized carbon nitride materials. The results from XPS analysis (Figures 3 and S4, see Supporting Information) support the presence of basic units of the tri-s-triazine ring, which is connected by the N atoms to form a π-conjugated polymeric network.^59^ The tri-s-triazine is also an integral unit of the melon-like structure of gCN. The C 1s spectra of all the different gCN samples consist of two peaks with binding energy values around 285.4 (C1) eV and 282.05 eV (C2), which can be associated with graphite carbon and 3-fold trigonal coordinated carbon by nitrogen atoms, respectively. The peak at around 282 eV suggests the presence of carbide-like species or specific carbon environments such as surface contaminants. The N 1s peaks appeared asymmetric in shape, and upon deconvolution, two peaks at around 396 eV (N1) and 398 eV (N2) were obtained. These peaks can be assigned to sp^2^- and sp-bonds between nitrogen and carbon atoms. A shoulder peak at a higher binding energy, 400 eV (N3), was assigned to sp-bonded nitrogen in the terminal C–N groups or the positive charge localization. The peaks for O 1s (O1) can be attributed to surface-adsorbed oxygen due to atmospheric exposure.^60^ Melamine has a higher nitrogen content (Table 1) and a preformed triazine structure, resulting in a more condensed and ordered polymeric gCN network with enhanced π-conjugation, leading to a more condensed gCN network compared to the uCN samples. This increased delocalization improves charge screening and results in slightly lower binding energies for both C 1s and N 1s signals, as confirmed by XPS fitting of melamine-derived precursors.^61,62^ Also, the shifts toward lower binding energy of mCN samples may be attributed to fewer oxidative defects (like C=O or N–O species), due to which the atoms remain in a lower oxidation state, causing a downward energy shift. Similarly, the higher binding energy values for uCN imply the presence of more oxidative byproducts, thus increasing binding energy. This can also be correlated with the higher content of oxygen in the case of elemental analysis (Table 1). Melamine favors a higher proportion of sp^2^ hybridized nitrogen (C=N–C) bonds, which contributes to a more conductive and electron-rich system. In contrast, uCN typically presents higher binding energies in the N 1s region. This aligns with more abundant terminal –NH_2_ or −NH groups, known to act as electron-withdrawing species and reduce local electron density.^63^ Additionally, urea-derived materials often exhibit more pronounced O 1s signals, indicative of surface oxidation or oxygen-containing defects introduced during decomposition or atmospheric exposure (Table 1). It is worth noticing that the rCN samples have the binding energy values shifted slightly toward lower values than their pCN counterparts. When the pCN samples are thermally heated, it causes the removal of oxidative defects (C=O and N–O), leading to a higher electron density, which is attributed to the lower shifts in binding energy.

**Figure 3:**
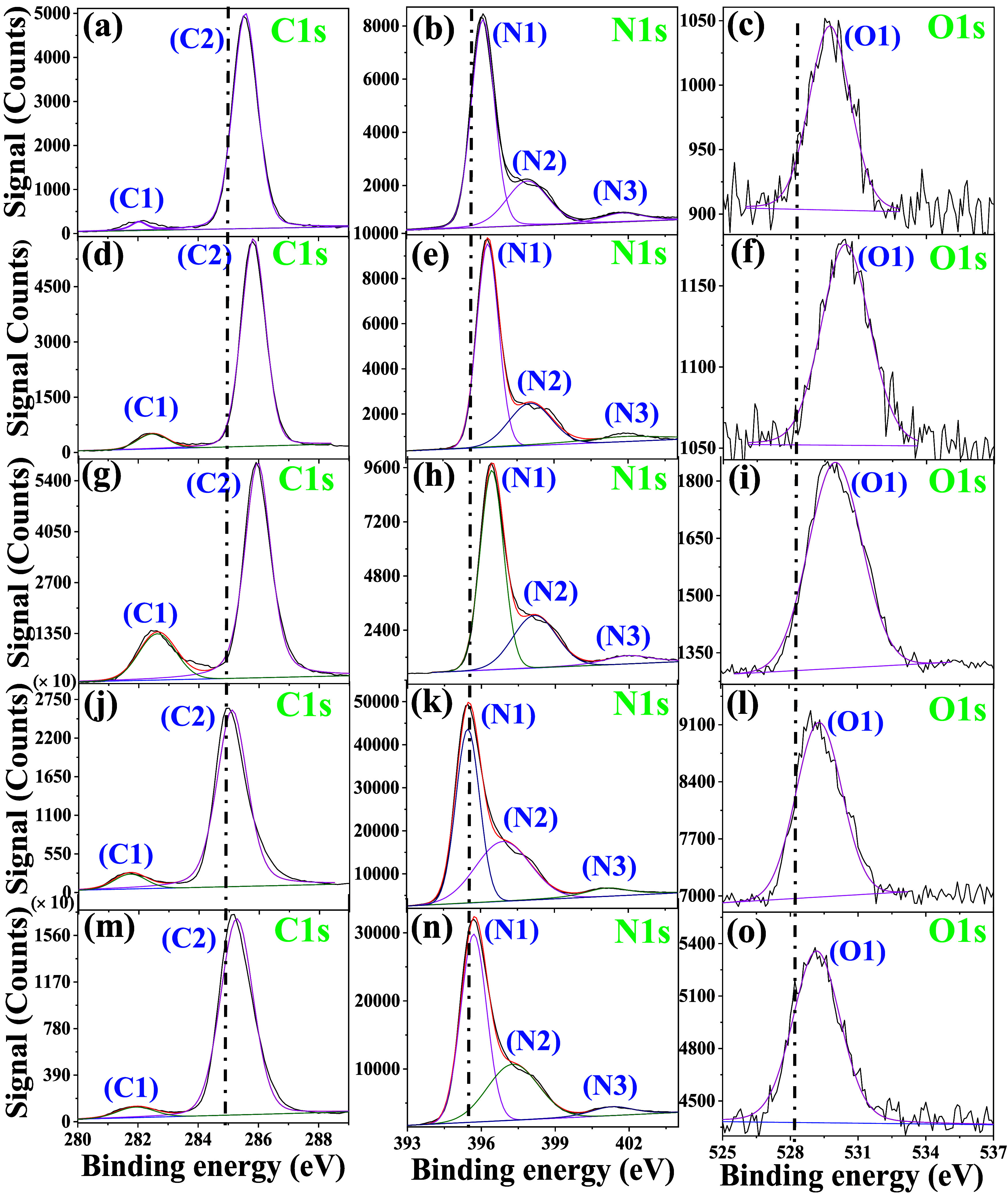
Core-level XPS spectra for CN (a–c), u_p_CN (d–f), u_r_CN (g–i), m_p_CN (j–l), and m_r_CN (m–o). The different peak positions in these core-level spectra are abbreviated as C1, C2, N1, N2, N3, and O1.

Altogether, the observed shifts in binding energies across the C 1s, N 1s, and O 1s spectra reflect a delicate interplay among precursor chemistry, degree of polymerization, surface functionalization, and postsynthesis treatments, highlighting how these parameters can be harnessed to tune the electronic properties of carbon nitride materials for advanced applications.^9^

To evaluate the effects of crystallinity and the role of defects of the melon-like structure (influenced by the choice of precursor and method of synthesis), we have performed galvanostatic experiments as well as DFT studies for lithium–sulfur batteries. One of the important applications of graphitic nitride is its use in lithium–sulfur batteries (Li–S) due to its high nitrogen content, which provides abundant active sites for lithium polysulfides (LiPSs) immobilization and promotes electrochemical reactions, as well as its ability to homogenize lithium-ion deposition and suppress dendrite growth.^64^

We investigated the interactions of LiPSs and the substrate to gain deeper insight into the role of reduced surfaces in mitigating the polysulfide shuttle effect in Li–S electrodes during the discharge process. The most stable configurations of LiPSs adsorbed on pristine and reduced mg-CN and hg-CN substrates are presented in Figures 4 and S5 (See Supporting Information), respectively. The adsorption energies of Li_2_S*_n_* (*n* = 1, 2, 4, 6, 8) and S_8_ species on the surface of pristine and reduced gCN substrates are presented in Figure 5b. Moreover, the adsorption energies of both reduced melon (rmg-CN) and heptazine (rhg-CN) substrates range from 1.83 to 4.90 eV, which are significantly higher than those of pristine analogues (1.17–2.98 eV), except for Li_2_S_8_ and Li_2_S_6_ species on gCN. Overall, this indicates that the introduction of vacancies enhances the interaction between LiPSs and the gCN (for both heptazine and melon-like models) substrate. For comparison, it is noteworthy that LiPSs exhibit the strongest adsorption on the rmg-CN substrate, suggesting that vacancies in the melon-like structure of gCN can effectively suppress LiPS shuttling in Li–S batteries.

**Figure 4:**
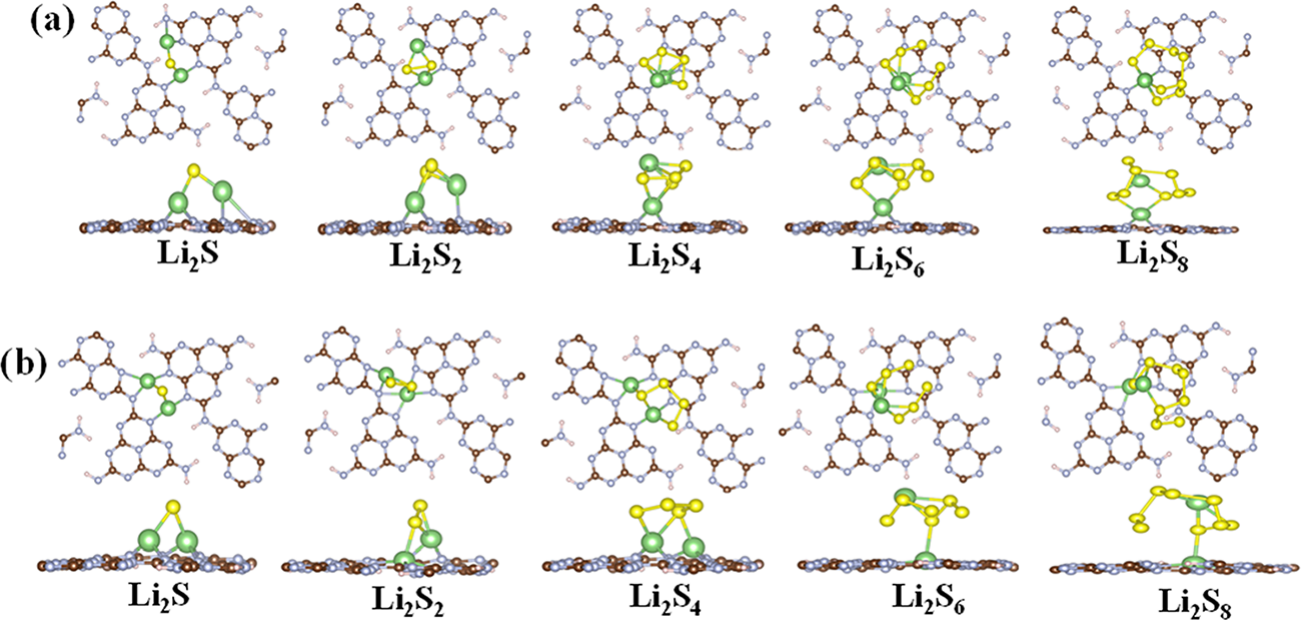
DFT optimized structures of adsorption lithium polysulfide molecules adsorbed on melon-like (a) pristine gCN and (b) reduced gCN. The green and yellow illustrate lithium and sulfur, respectively.

**Figure 5:**
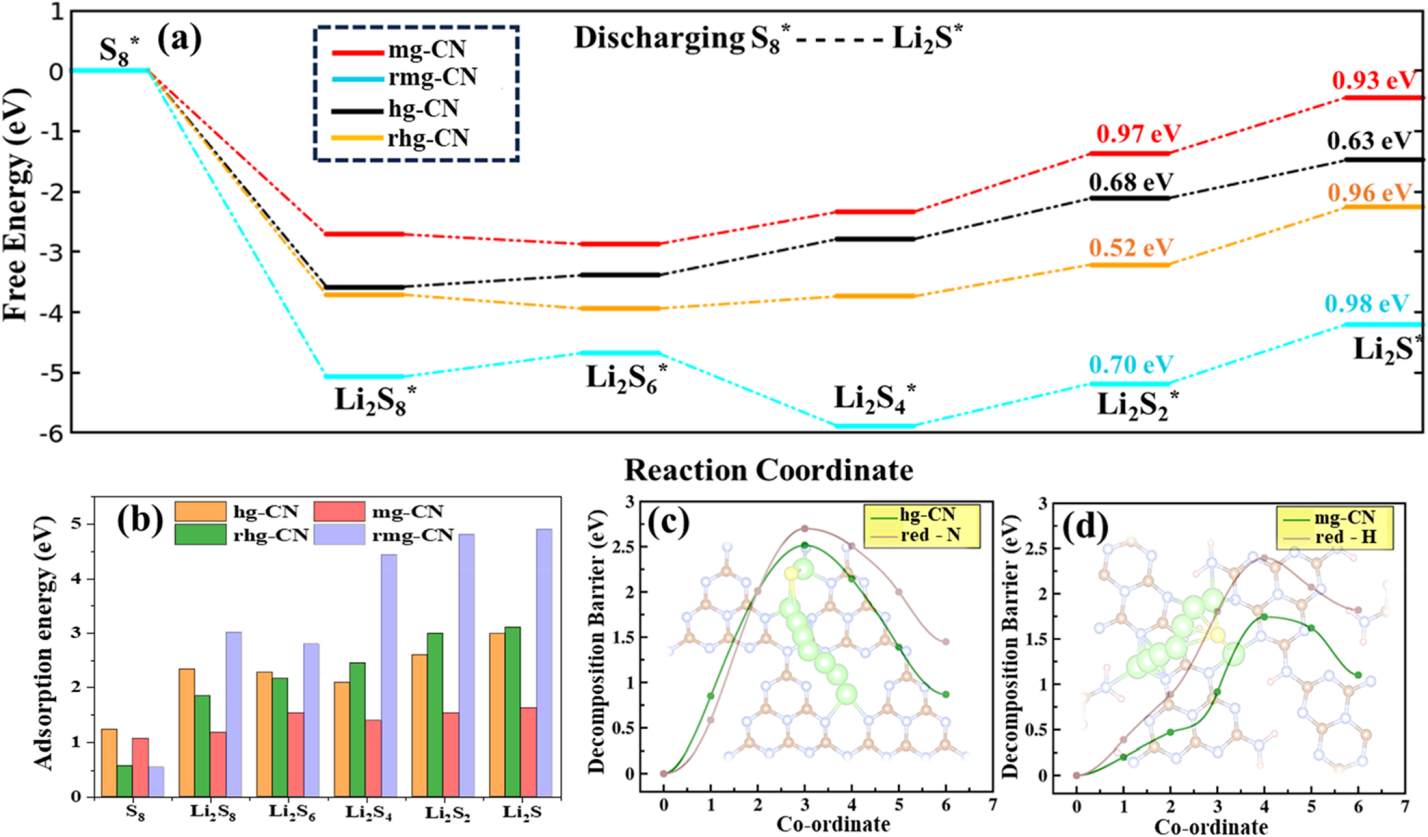
(a) Calculated Gibbs free energy diagram and (b) adsorption energies of sulfur reduction reaction in pristine and reduced structures of melon-like (mg-CN, rmg-CN) and heptazine-based (hg-CN, rhg-CN) models. Decomposition barrier of Li_2_S for pristine and reduced structures of (c) heptazine-based and (d) melon-like models. Red-N and red-H refer to the reduced structures where a nitrogen and a hydrogen are removed, respectively.

To evaluate the discharge performance of the electrodes, we investigated sulfur reduction reactions on both pristine and reduced gCN substrates. The Gibbs free energy of each intermediate reaction step from S_8_* to Li_2_S* on both pristine and reduced rhg-CN and mg-CN substrates is shown in Figure 5a. For both systems, the initial step, corresponding to the reduction of S_8_ to Li_2_S_8_, is exothermic regardless of the substrate, indicating the spontaneous nature of this conversion. Our calculations reveal that for pristine hg-CN and mg-CN, the rate-limiting step is the transition from Li_2_S_4_ to Li_2_S_2_. However, in the reduced substrates, this energy barrier is further lowered, making the Li_2_S_2_ to Li_2_S transition the new rate-limiting step.

Additionally, the decomposition barriers of Li_2_S play a crucial role in enhancing the oxidation reaction kinetics, thereby extending the lifecycle of the electrode. To gain deeper insights, we investigated the decomposition mechanisms of Li_2_S oxidation (Li_2_S → LiS + Li^+^ + e^–^**) and energy profiles along the optimal reaction pathways for Li_2_S decomposition, which are illustrated in Figure 5c,d. The decomposition barriers for Li_2_S on pristine substrates (2.51 eV for hg-CN and 1.74 eV for mg-CN) are significantly lower than those on the rgCN substrates (2.70 eV for rhg-CN and 2.39 eV for rmg-CN). A similar trend is also observed in graphene and its reduced counterpart.^65^ In summary, the melon-like structure of gCN appears to be a more promising substrate for effectively suppressing LiPS shuttling and promoting redox reactions in Li–S batteries.

Based on the results of DFT calculations, the electrochemical behavior of gCN-based cathodes was evaluated in Li–S batteries. Before exploring these studies, we recorded SEM images of different gCN/S composites to explore the morphology of these systems after sulfur infusion. From Figure S6 (see Supporting Information), the morphology of the different gCN supports changes slightly in comparison to the bare supports, due to the infusion of the sulfur into the gCN moiety. Figure 6a displays the rate capabilities at varying C-rates, revealing that the melamine-derived (both reduced and pristine) electrode achieves the highest specific capacity at all charge/discharge rates (0.1C–1C). This can be attributed to its better crystallinity, as can be seen from XRD and SEM images, leading to improved sulfur utilization. The increased number of active sites in m_p_CN and m_r_CN, as evident from analytical (Raman and XPS) and computational studies, enhances polysulfide adsorption and catalytic activity.

**Figure 6:**
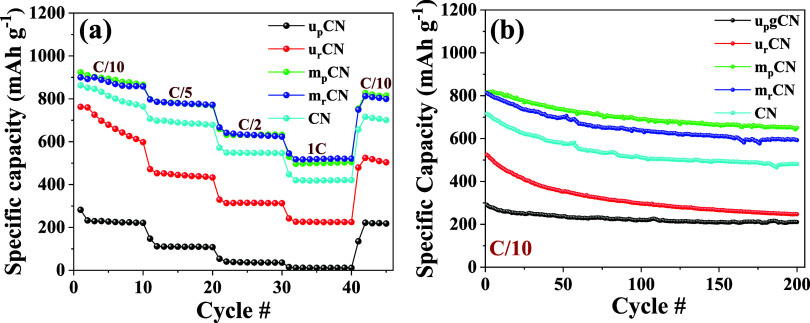
Electrochemical performance of Li–S cells using cathodes with 75 wt % sulfur in the gCN/S composite of different samples. (a) Charge/discharge specific capacity at various C-rates (0.1C, 0.2C, 0.5C, and 1C). (b) Capacity retention at long cycles of different graphitic carbon nitride/sulfur composites at 0.1C.

Capacity retention studies (Figure 6b) show that pristine melamine-derived electrodes retain a higher capacity over extended cycling compared with reduced counterparts, with m_p_CN being the best among all. While the defects in m_r_CN provide capacity enhancement during fast charge/discharge cycles, the higher oxygen content in the m_p_CN sample provides more sites for chemical confinement of the polysulfides, preventing the participation in the shuttle effect phenomena, thus resulting in better capacity retention over extended cycling.^66^ The capacity retention rates (%) and specific capacities after the 200th cycle for these samples are presented in Table 2. The m_p_CN sample was further studied for long-term cycling performance (500 cycles). Coulombic efficiency and specific charge/discharge capacities (Figure S7­(a), see Supporting Information) further emphasize the capacity retention of m_p_CN (500 cycles at 0.1C). It is noteworthy to mention that the improved performance of Li–S batteries based on these gCN/sulfur composite cathode was achieved only through precursor selection and vacuum-thermal reduction process, which matches or exceeds the performance data reported at lower C-rate (ex. 0.05C^67^) or cathodes which were produced through more complex post-processing methods such as magnesiothermic denitriding,^68^ oxygenation at high temperatures (ex. 900 °C^61^), or presence of transition metal catalysts.^58,69,70^ The voltage profiles of m_p_CN (Figure S7b, see Supporting Information) display consistent charge/discharge plateaus which correspond to different stages of sulfur and lithium reactions,^66^ indicating good reversibility, although capacity degradation is noticeable in later cycles. This trade-off between initial capacity and retention is directly correlated with the defect concentration and oxygen content, as highlighted in the Raman, XPS, and elemental analyses. Upon comparison of oxygen percentages and defect concentration from Raman (*I*_D_/*I*_G_) (Table 2), it can be affirmed that the lower the oxygen content and the higher the defect concentration, the better the average capacity of the samples at 0.1C.

**Table 2:** Details of Oxygen%, Defect Concentration (*I*_D_/*I*_G_), and Specific Capacity and Capacity Retention Rate (%) at the 200th Cycle at 0.1 C for the Different gCN Samples

sample	oxygen at. %	*I*_D_/*I*_G_	specific capacity of the 200th cycle at 0.1 C (mAh g^–1^)	capacity retention rate (%) at 200th cycle at 0.1 C
CN	1.2	0.83	481.09	68.3
m_p_CN	1.2	1.05	649.98	79.4
m_r_CN	0.9	1.08	592.66	74.0
u_p_CN	8.1	0.94	210.49	74.6
u_r_CN	2.6	1.01	246.74	48.4

## Conclusions

The results from structural analyses collectively affirm the dominance of the melon-like framework in the synthesized gCN samples. The thorough investigation of the obtained XRD patterns for the synthesized samples with different models for graphitic carbon nitride further supports the clear alignment with the *P*2_1_2_1_2 symmetry and reinforces that the melon-like structure is maintained, regardless of precursor choice or reduction treatment. The synthesis of gCN from different precursors (urea and melamine) leads to variations in crystallinity, defect concentration, and surface area, all of which influence the electrochemical behavior. The deconvoluted C 1s and N 1s peaks from XPS spectra indicate a higher proportion of sp^2^ hybridized nitrogen in the melamine-based samples, suggesting a more condensed network with the presence of tri-s-triazine units, characteristic of the melon structure. In contrast, the urea-based samples exhibit an increased oxygen content and more oxidative defects, contributing to their lower crystallinity. The elevated *I*_D_/*I*_G_ ratio in reduced samples, particularly reduced melamine (m_r_CN), confirms the creation of more disordered carbon sites, which seemed beneficial for enhancing sulfur adsorption, as reflected in the experimental as well as in the DFT studies. Pristine melamine-based gCN (m_p_CN) exhibited superior capacity retention along with better specific capacity comparable to its reduced counterpart, indicating its structural stability over prolonged cycling. The DFT calculations further support these results, revealing lower Gibbs free energy and adsorption energies for sulfur species on reduced gCN surfaces of the melon-like network. This study provides valuable insights into the structure–property relationship of gCN and offers a promising approach for developing advanced materials for Li–S batteries.

## Supplementary Material



## Notes

Comparative XRD patterns of synthesized gCN with triazine, heptazine (literature and simulated) models; SEM images of the different gCN/S composites; pore size distribution curves and N_2_ adsorption–desorption of different gCN samples; survey XPS spectra for CN, u_p_CN, u_r_CN, m_p_CN, m_r_CN; DFT optimized structures of melon-like and heptazine (pristine and reduced) models; DFT optimized structure of adsorption of Lithium polysulfides on heptazine models (pristine and reduced); Specific charge/discharge capacity and Coulombic efficiency of the m_p_CN cathode; Voltage profiles of the m_p_CN cathode during charge/discharge at the first, 50th, 100th, 200th, and 500th cycles (PDF)

## Abbreviations

gCN: graphitic carbon nitride
_p_CN: pristine graphitic carbon nitride
_r_CN: reduced graphitic carbon nitride
mCN: melamine-derived graphitic carbon nitride
uCN: urea-derived graphitic carbon nitride
m_p_CN: melamine-derived pristine graphitic carbon nitride
m_r_CN: melamine-derived reduced graphitic carbon nitride
u_p_CN: urea-derived pristine graphitic carbon nitride
u_r_CN: urea-derived reduced graphitic carbon nitride
mg-CN: pristine graphitic carbon nitride with melon-like structure
rmg-CN: reduced graphitic carbon nitride with melon-like structure
hg-CN: pristine graphitic carbon nitride with heptazine structure
rhg-CN: reduced graphitic carbon nitride with heptazine structure
LiPSs: lithium polysulfides
red-N: reduced C_3_N_4_ with N atom being removed
red-H: reduced C_3_N_4_ with H atom being removed
