# Supplementary material for: From Structure to Functional Implications: Investigation of the Melon-Like Framework of Graphitic Carbon Nitride for Li–S Batteries
Source: ACS Omega. 2025 Sep 25;10(40):47375–85. doi: 10.1021/acsomega.5c06720 (PMC12529386; doi:10.1021/acsomega.5c06720)
Supplement: Supplementary file 1 [file ao5c06720_si_001.pdf]

Supporting Information

From Structure to Functional Implications: Investigation of the Melon-Like Framework of Graphitic Carbon Nitride for Li-S Batteries

Jyoti Pandey,[a] Aliakbar Yazdani,[b] Mukesh Jakhar,[a,c] Valeri Petkov,[a] Veronica Barone,[a] Chi-Hao Chang,[d] Gabriel Caruntu,[b] Yi Ding,[e] and Bradley D. Fahlman[b]*

[a]Department of Physics, Central Michigan University, Mt. Pleasant, MI 48859, USA

[b]Department of Chemistry and Biochemistry, Central Michigan University, Mt. Pleasant, MI 48859, USA

[c] Inter-University Accelerator Center, Aruna Asaf Ali Marg, New Delhi, India

[d]The Dow Chemical Company, Dow Performance Silicones, 5300 11 Mile Road, Auburn, MI 48611, USA

[e]U.S. Army Combat Capabilities Development Command (DEVCOM) - Ground Vehicle Systems Center (GVSC), Warren, MI 48397, USA

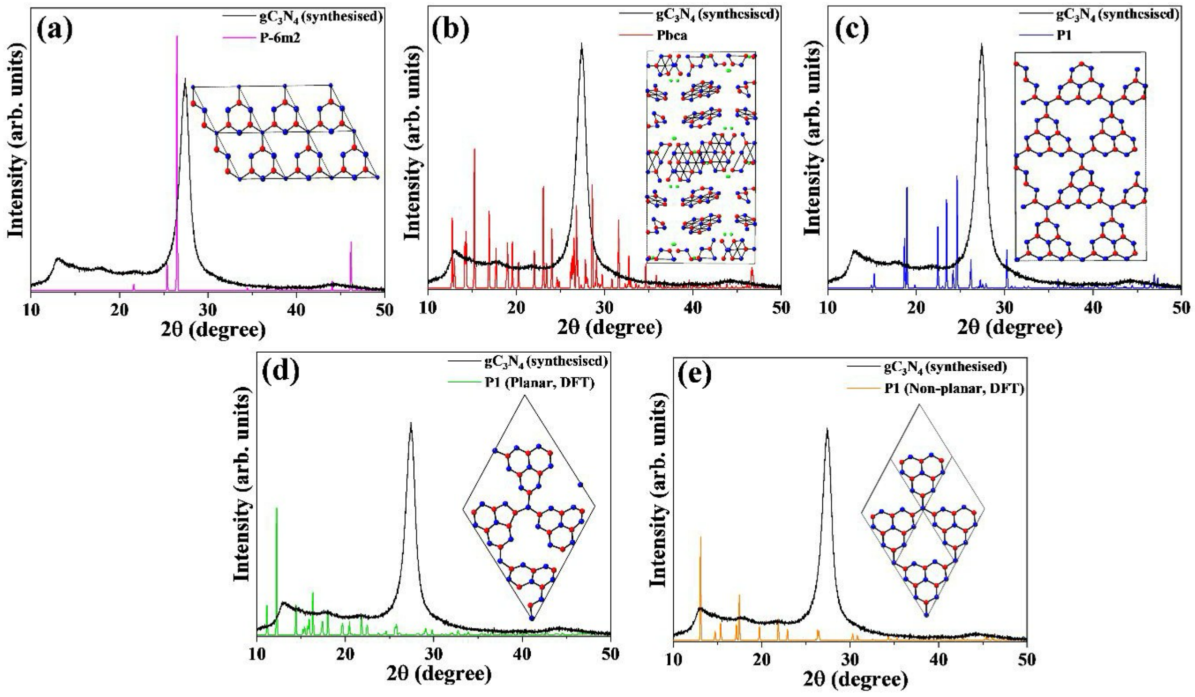

Figure S1. Comparison of XRD pattern of synthesized gCN with **(a)** triazine model ($P\bar{6}m2$)[11]; heptazine models **(b)** Pbca[21], **(c)** P1[21] and structure simulated by DFT considering the heptazine model, **(d)** P1 (planar), **(e)** P1 (non-planar). Insets includes the projection of the respective crystal structure of gCN. C, N and H are represented in red, blue and green, respectively.

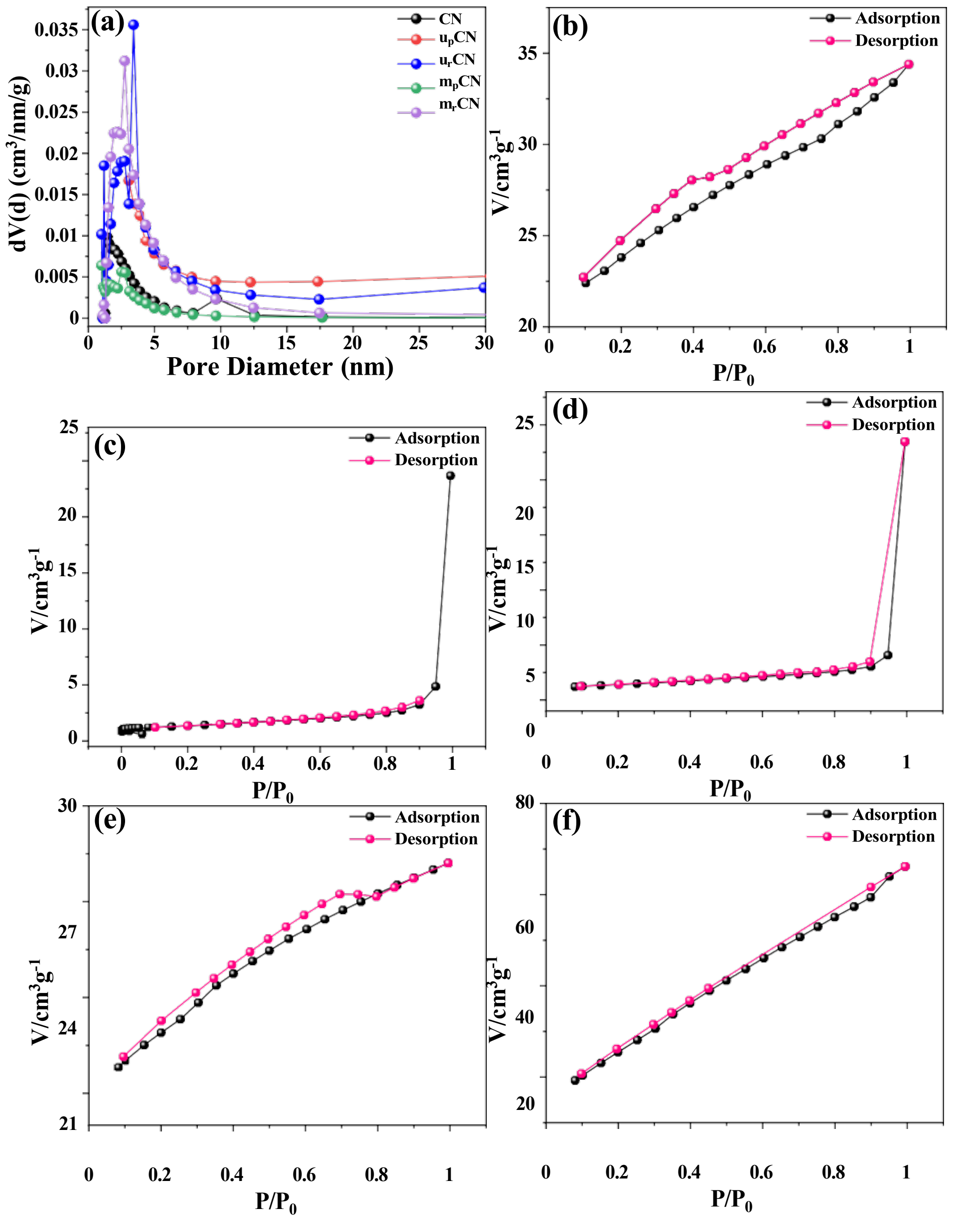

Figure S2. (a) Pore size distribution curves of different gCN samples. N_2 adsorption-desorption isotherms for **(b)** CN, **(c)** u_pCN, **(d)** u_rCN, **(e)** m_pCN, and **(f)** m_rCN.

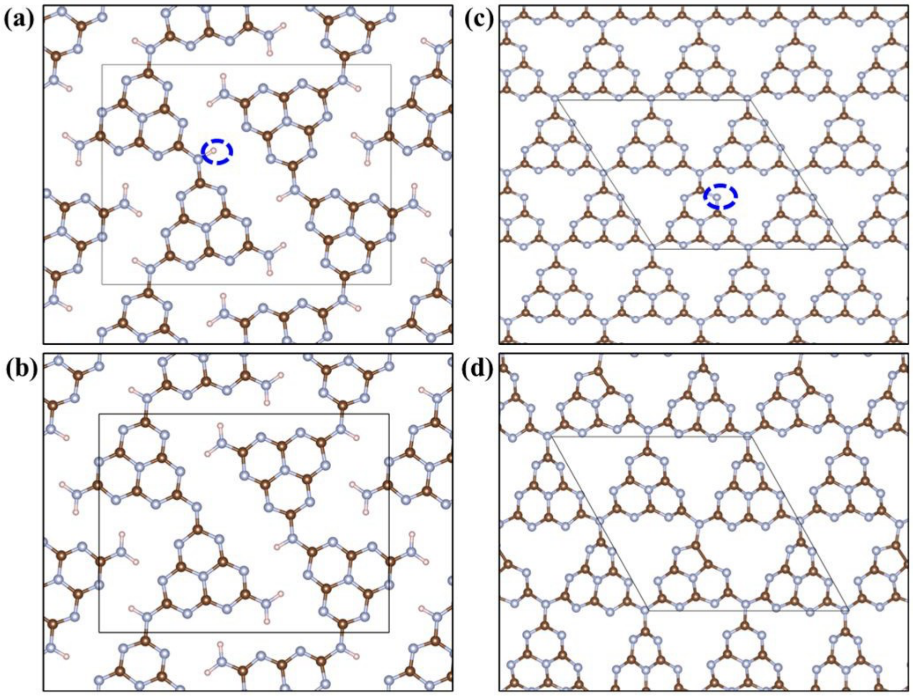

Figure S3. DFT optimized structures of **(a)** Melon-like pristine gCN, **(b)** Melon-like reduced gCN, **(c)** Heptazine-based pristine gCN, and **(d)** Heptazine-based reduced gCN. The blue dotted circles represent the site that is most favorable for reduction as per energetics by DFT. C, N, and H are represented in brown, light blue, and light pink, respectively.

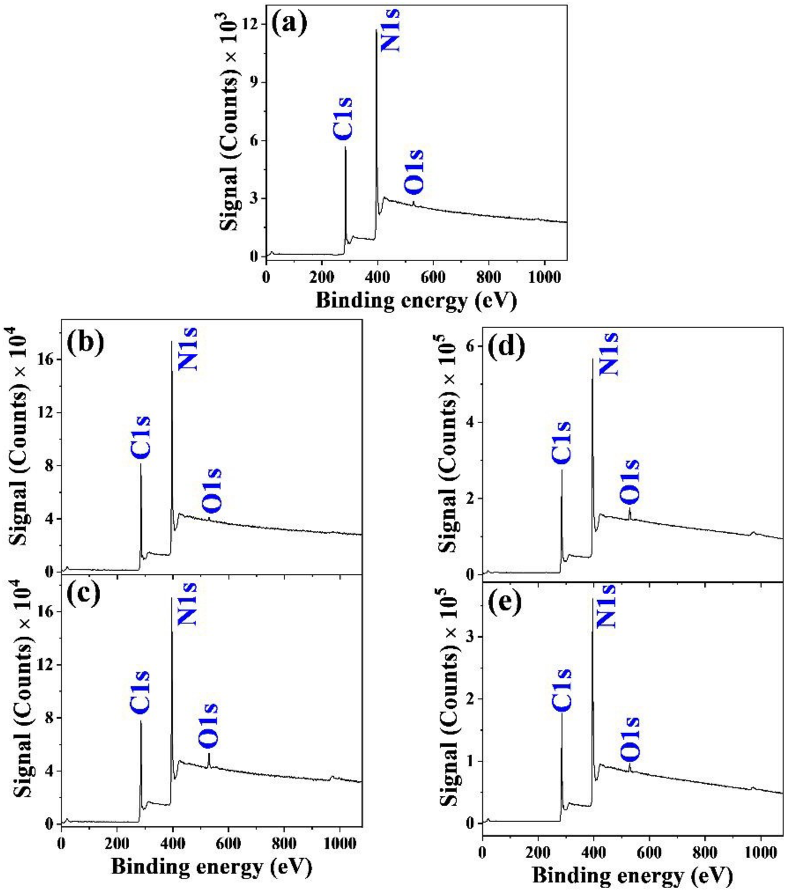

Figure S4. Survey XPS spectra for the **(a)** CN, **(b)** u_pCN, **(c)** u_rCN; **(d)** m_pCN, and **(e)** m_rCN.

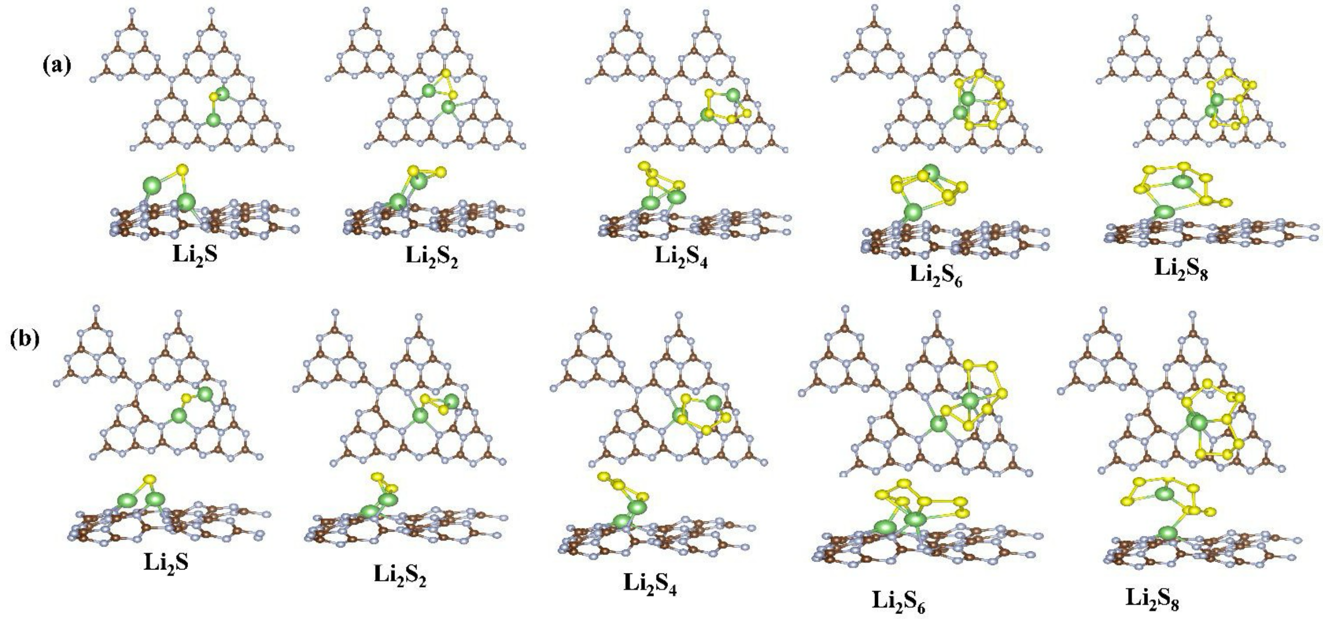

Figure S5. DFT optimized structures of adsorption lithium polysulfides molecules adsorbed on heptazine based **(a)** pristine gCN, and **(b)** reduced gCN. The green and yellow indicate lithium and sulfur, respectively.

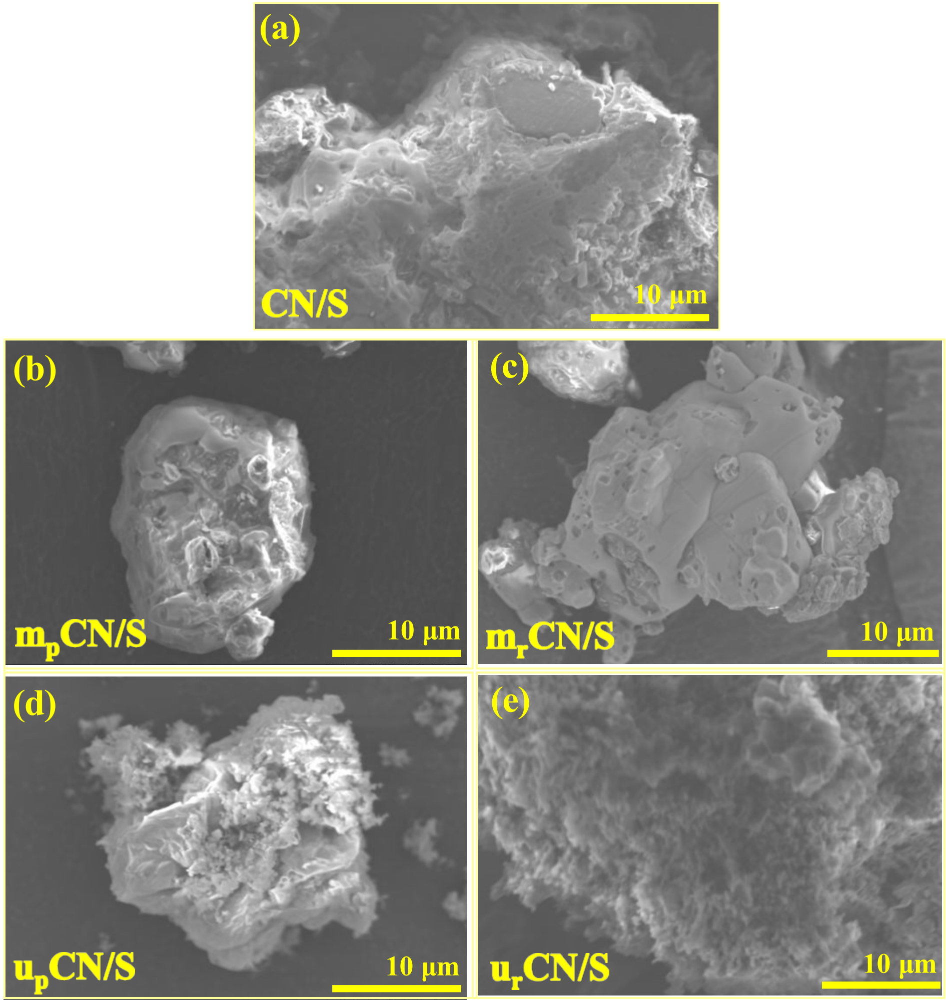

Figure S6. SEM images of different gCN composites with S; **(a)** Commercial gCN/S **(b)** m_pCN/S, **(c)** m_rCN/S, **(d)** u_pCN/S, and **(e)** u_rCN/S. The images show that the morphology of the different gCN changes slightly in comparison to the bare supports, due to infusion of the sulfur into the gCN framework.

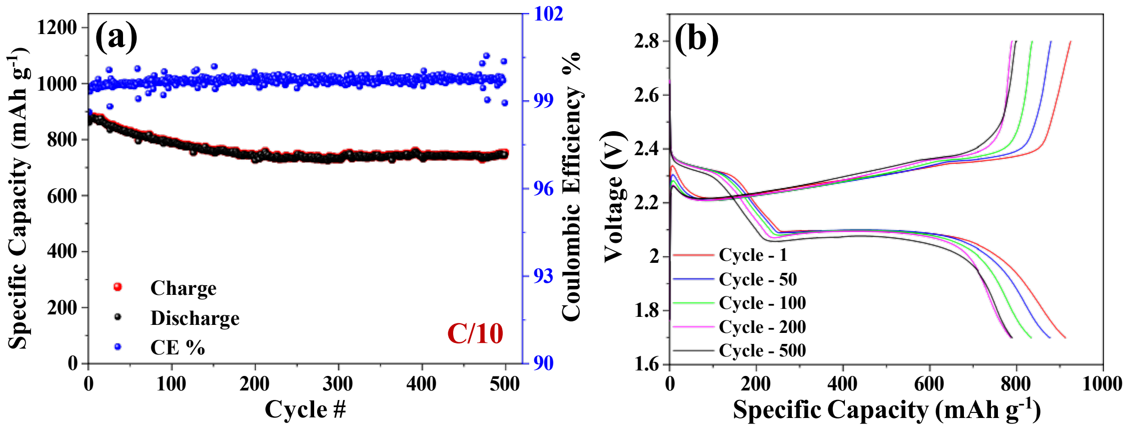

Figure S7. (a) Specific charge/discharge capacity & Coulombic efficiency. Average columbic efficiency of the 500 cycles is 99.7%, and **(b)** voltage profiles during charge/discharge at the 1st, 50th, 100th, 200th, and 500th cycles of the m_pCN/sulfur composite cathode.
